# Fault Diagnosis for Rotating Machinery Using Multiscale Permutation Entropy and Convolutional Neural Networks

**DOI:** 10.3390/e22080851

**Published:** 2020-07-31

**Authors:** Hongmei Li, Jinying Huang, Xiwang Yang, Jia Luo, Lidong Zhang, Yu Pang

**Affiliations:** 1 School of Big data, North University of China, Taiyuan 030051, China; hongmeili@tyust.edu.cn (H.L.); yangxw@nuc.edu.cn (X.Y.); 2 School of Mechanical Engineering, North University of China, Taiyuan 030051, China; sjj1314@nuc.edu.cn (J.L.); ldzhang@163.com (L.Z.); pangyu@163.com (Y.P.)

**Keywords:** multiscale permutation entropy, information fusion, multi-channel, convolutional neural networks, fault diagnosis, rotating machinery

## Abstract

In view of the limitations of existing rotating machine fault diagnosis methods in single-scale signal analysis, a fault diagnosis method based on multi-scale permutation entropy (MPE) and multi-channel fusion convolutional neural networks (MCFCNN) is proposed. First, MPE quantitatively analyzes the vibration signals of rotating machine at different scales, and obtains permutation entropy (PE) to construct feature vector sets. Then, considering the structure and spatial information between different sensor measurement points, MCFCNN constructs multiple channels in the input layer according to the number of sensors, and each channel corresponds to the MPE feature sets of different monitored points. MCFCNN uses convolutional kernels to learn the features of each channel in an unsupervised way, and fuses the features of each channel into a new feature map. At last, multi-layer perceptron is applied to fuse multi-channel features and identify faults. Through the health monitoring experiment of planetary gearbox and rolling bearing, and compared with single channel convolutional neural networks (CNN) and existing CNN based fusion methods, the proposed method based on MPE and MCFCNN model can diagnose faults with high accuracy, stability, and speed.

## 1. Introduction

Rotating machinery is widely used in many fields of heavy industry and plays an important role in modern industrial production [1]. Gears and rolling bearings are the core components used to support the rotating body and transmit torque and power. They play a vital role in the transmission system. Any fault of bearing and gear may lead to unnecessary shutdown, leading to significant economic loss and even casualties [2,3]. Therefore, real-time health monitoring and fault diagnosis are very important for the safe operation of machinery.

Vibration signal analysis is the most commonly used diagnostic method in mechanical fault diagnosis at present [4]. In recent years, many researchers have proposed to use information fusion method for fault diagnosis of rotating machinery. Meghdad et al. [5], Loutas et al. [6], Lei et al. [7], and Liu et al. [8] used the signal processing technology to extract features from different signal sources and fuse them. Then, artificial neural network (ANN), independent component analysis (ICA), adaptive fuzzy neural inference system, and relevance vector machine (RVM) classification algorithm were used to classify the fused features, respectively. Meghdad et al. [9], Peng et al. [10], and Jaramillo et al. [11] adopted D-S evidence theory and Bayesian reasoning method for decision fusion, respectively. Although the information fusion methods in the above literatures improve the accuracy of fault diagnosis, they have certain limitations in feature extraction, mainly in the following aspects: (1) In information fusion, they only extract multiple types of features from the signals collected by the single sensor and fuse them, without analyzing the sensor information of other monitoring points. (2) the information after multi-sensor fusion presents the characteristics of big data such as mass, multi-source, heterogeneity, complexity, and real-time. Traditional intelligent diagnostic algorithms have poor nonlinear fitting ability, which is difficult to meet the requirements of big data.

In order to solve the above drawbacks, convolutional neural networks (CNN) may provide effective solutions for intelligent information fusion and fault diagnosis. CNN is a model of deep learning, which uses end-to-end processing technology to achieve feature extraction and fault classification [12]. In recent years, some researchers proposed to use CNN for fault diagnosis [13,14,15,16,17]. Jing et al. [13], Xia et al. [14], and Sun et al. [15] spliced multiple signals into one-dimensional or two-dimensional matrices, and used CNN to extract features for fault recognition. Han et al. [16] constructed dynamic ensemble convolutional neural networks (DECNN), Jiang et al. [17] proposed multi-scale convolutional neural networks (MSCNN), and in [18], we proposed CNNEPDNN model. These models have a common feature, that is, the model contains several parallel feature learning branches. The inputs of each branch are constructed according to a certain standard, and the extracted features of each branch are fused through the network layer to complete the model training. The fusion method based on CNN can capture fault information of different scales and improve the accuracy of diagnosis, but some challenges still exist: (1) The fusion mode based on data splicing not only increases the number of input samples, but also leads to the slow convergence due to the difference of original data [19]. (2) The structure of CNN based on feature fusion mode is cumbersome and characterized by many training parameters, long training time, and slow convergence.

Multi-channel CNN is widely used in the fields of image processing and speech recognition. In these fields, the input can be naturally divided into different channels, such as the color channel of the image and the wavelength of the speech [19]. Liu et al. [20] constructed a multi-channel CNN target detection framework. First, mid-wave infrared image (MWIR), visible image (VI) and motion image were fused in an unsupervised way to generate a BGR style three-channel image, which was used as the input of multi-channel CNNs. The experimental results show that the proposed approach improves the recognition accuracy, implementation simplicity and low computation complexity. Kato et al. [21] proposed three different multi-channel CNN frameworks for image super-resolution, namely, the architecture with four parallel CNNs (4P), the single CNN architecture with four channels (4CH) and the four channels CNN with rotary averaging technique (4CH-R). Experimental results show that the processing speed of 4CH was the fastest with few parameters, the peak signal-to-noise ratio (PSNR) of 4CH-R was the highest, thus verifying the practicability of multi-channel CNN architecture. Liu et al. [22] constructed a multi-channel CNN architecture for language sentiment analysis. First, three input channels were constructed in CNN, and each channel consists of one embedding layer, one convolution layer, and one pooling layer. Then the Chinese text was converted to pinyin, characters and words, and fed into three channels, respectively. Finally, the features extracted from each channel were concatenated to feed into a fully connected dense layer, and the analysis results were output through the output layer. Inspired by multi-channel CNN processing image and speech, a multi-channel fusion CNN (MCFCNN) model based on CNN and data information fusion is proposed. MCFCNN uses a multi-channel structure to achieve information complementarity between multiple sensors, improve the fault diagnosis rate, and meet the real-time requirements of fault diagnosis. Different from the above multi-channel CNN, the backbone of MCFCNN is a traditional CNN structure, that is, a single CNN structure contains multiple channels, and the corresponding input data of each channel is independent of each other.

Nevertheless, the structure of mechanical equipment is very complex, and the interaction and coupling effect between the components make the vibration signal contain many inherent oscillation modes on different time scales [23]. Traditional CNN structure could not capture these inherent multi-scale features due to their lack of multi-scale feature extraction capabilities [17]. The multi-scale nonlinear analysis method proposed in recent years is proposed to extract fault features from multiple time scales to improve the fault diagnosis rate and achieve good results. For example, Zhang et al. [24] used multi-scale entropy (MSE) to extract fault features from bearing vibration signals and input them to the adaptive neural fuzzy inference system (ANFIS), good diagnostic results were obtained. Yan et al. [25] first used the improved multi-scale discrete entropy (MDE) to extract the fault feature from the vibration signal of rolling bearing, then used the max-relevance min-redundancy (mRMR) algorithm to select the sensitive feature from the multi-scale feature, and input them into the extreme learning machine (ELM) for classification. Zhao et al. [26] decomposed the vibration signals of rolling bearings into a set of sub-band signals through wavelet packet decomposition (WPD), and calculated the multi-scale permutation entropy (MPE) values of all sub-band signals to form a feature vector, and then used HMM to identify the fault. Wu et al. [27] combined MPE and support vector machine (SVM) method for bearing fault diagnosis. Experimental results show that compared with methods based on single-scale permutation entropy (PE) and MSE, the feature extraction method based on MPE has higher prediction accuracy. Moreover, MPE is more robust to the change of training sample size. In addition, some studies [25,28] have found that compared with MSE and MDE, MPE has faster calculation speed. Therefore, this paper uses MPE method to analyze the complexity of vibration signals on multiple scales, and feeds the MPE value to MCFCNN.

In MCFCNN, multiple independent channels are established in the input layer, and each channel corresponds to the MPE feature set of each measuring point sensor. Each convolutional kernel further learns the deep features of each channel separately, and fuses the learned feature maps of each channel into a new feature map [29]. MCFCNN uses multi-channel instead of parallel network structure to input data from different measurement points at the same time to obtain more fault information. Moreover, multiple channels share a CNN structure, which effectively reduces the number of parameters and training time, and improves the real-time of fault diagnosis. The main contributions of this study can be summarized as follows: (1) Applying MPE to gearbox and bearing fault diagnosis; (2) Design a new intelligent fusion model MCFCNN, which provides an idea for the same type of sensor fusion. (3) The proposed method is used for bearing and gearbox health status data, and compared with single-channel CNN and existing CNN-based fusion methods, MCFCNN model has faster convergence speed and higher classification accuracy.

The remainder of this paper is organized as follows. In Section 2, the basic theory of MPE and CNN are elaborated. In Section 3, the proposed MCFCNN model based on CNN and multi-sensor data fusion methods is introduced. In Section 4, the test rig and experimental datasets for planetary gearboxes and rolling bearing are described, and the proposed method is verified by the comparison with other diagnostic methods, and the results of the model under different experiments are discussed. The conclusions are drawn in Section 5.

## 2. Basic Theory

### 2.1. Multiscale Permutation Entropy

**Figure 1:**
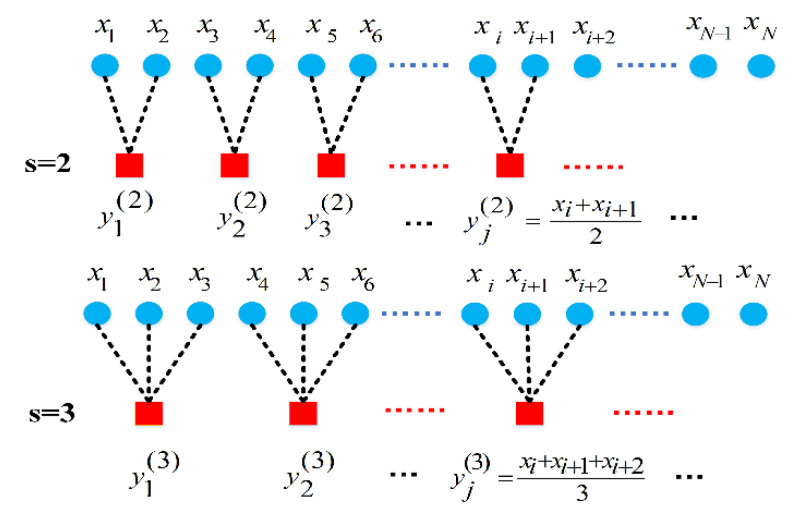
The coarsening process of the time series with scale factors s = 2 and s = 3.

Aziz et al. [30] proposed multi-scale permutation entropy (MPE) based on the research of PE [31] method and multiscale analysis [32]. Its basic idea was to coarsen the time series at multiple scales, and then calculate the PE of the coarsening sequence. The calculation process is as follows:

Step 1: Constructing the coarse-grained time series. Given a time series $X{=\{x}_{1}{,x}_{2},\ldots {,x}_{N}\}$ of length N, the coarse-grained time series is constructed by time scale factor s. The coarsening process of the time series with scale factors s = 2 and s = 3 are shown in Figure 1. Using a window of length s to move over the original signal to compute the average to obtain the corresponding coarse-grained signal $\left\{y_{j}^{\left(s\right)}\right\}$. The length of the coarse-grained sequence is determined by the scale factor. When s = 1, the coarse-grained sequence degenerates to the original sequence; when s > 1, the original sequence becomes a coarse-grained sequence of length $\frac{N}{S}$. The calculation formula is as follows:

(1) $$\begin{array}{c} y^{\left(s\right)}(j)=\frac{1}{s}\overset{js}{\underset{i=(j-1)s+1}{\sum }}x(i)\\ j=1,2,\ldots ,\left\lfloor \frac{N}{s}\right\rfloor \end{array}$$

Step 2: Calculating PE value for coarse-grained time series $\left\{y^{\left(s\right)}(j)\right\}$. First, the phase space reconstruction technique is applied to map the coarse-grained sequence $\left\{y^{\left(s\right)}(j)\right\}$ into m dimensional data spaces.

(2) $$\left[\begin{array}{cccc} y^{\left(s\right)}(1) & y^{\left(s\right)}(1+\lambda ) & \ldots & y^{\left(s\right)}(1+(m-1)\lambda )\\ \ldots & \ldots & \ldots & \ldots \\ y^{\left(s\right)}(i) & y^{\left(s\right)}(i+\lambda ) & \ldots & y^{\left(s\right)}(i+(m-1)\lambda )\\ \ldots & \ldots & \ldots & \ldots \\ y^{\left(s\right)}(k) & y^{\left(s\right)}(k+\lambda ) & \ldots & y^{\left(s\right)}(k+(m-1)\lambda ) \end{array}\right]$$

where m is the embedded dimension and λ is the delay time, $k=\left\lfloor \frac{N}{s}\right\rfloor -(m-1)\lambda$ is the number of reconstruction vectors. Then m reconstruction components of $y^{\left(s\right)}(i)$ are arranged in ascending order.

(3) $$y^{\left(s\right)}(i+(j_{1}-1)\lambda )\leq y^{\left(s\right)}(i+(j_{2}-1)\lambda )\leq \ldots \leq y^{\left(s\right)}(i+(j_{m}-1)\lambda )$$

where $j_{1},j_{2},\ldots ,j_{m}$ represent the index of each element, if $y^{\left(s\right)}(i-(j_{1}-1)\lambda )=y^{\left(s\right)}(i-(j_{2}-1)\lambda )$ exists, it will be sorted according to the value of $j_{1}$ and $j_{2}$, when $j_{1}<j_{2}$, there is $y^{\left(s\right)}(i-(j_{1}-1)\lambda )\leq y^{\left(s\right)}(i-(j_{2}-1)\lambda )$. Therefore, any vector $y^{\left(s\right)}(i)$ can get a set of symbol sequences $\left(j_{1},j_{2},\ldots ,j_{m}\right)$. Assume that the probability of occurrence of each symbol sequence is $P_{j}$, the PE of $y^{\left(s\right)}(j)$ is calculated.

(4) $$H_{PE}^{\left(s\right)}=-\sum_{j=1}^{k}P_{j}InP_{j}$$

(5) $$H_{MPE}=[H_{PE}^{1},H_{PE}^{2},\ldots ,H_{PE}^{s}]$$

where, $H_{PE}^{\left(s\right)}$ represents the PE of coarse-grained time series $\left\{y^{\left(s\right)}(j)\right\}$, $H_{MPE}$ is an s-dimensional vector representing the PE of the time series X over multiple time scales.

### 2.2. Convolutional Neural Networks

**Figure 2:**
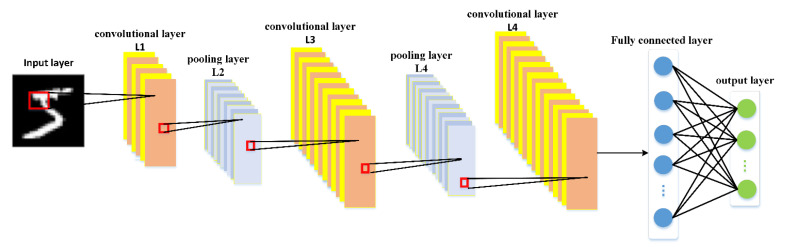
Structure of Lenet model.

Convolutional neural networks (CNN) is based on multi-layer supervised learning and it requires a large amount of labeled data to complete model training by repeating forward propagation and backward propagation. CNN is usually composed of feature extractor and multilayer perceptron (MLP). The feature extractor consists of multiple alternating pooling and convolutional layers. The convolutional layer is to extract the features from input data, whereas the pooling layer is to decrease the feature dimension [33]. The fully connected MLP is classified according to the features learned by convolution and pooling layers. Figure 2 shows one typical model of convolutional neural networks, called Lenet, designed by Y. LeCun et al. [34]. The Lenet model has one input layer, three convolution layers, two pooling layers, one fully connected layer and one output layer, in which the convolutional layer and the pooling layer are alternately connected.

The convolutional layer is responsible for feature extraction through a convolution kernel, which is essentially a weight matrix. The convolutional layer contains multiple convolution kernels with different weights. The convolutional layer slides different convolution kernels on the input data by sliding the window. The convolutional kernel and the corresponding area of the sliding window are convoluted to obtain local perception and the matrix obtained by the convolution operation after sliding is called feature mapping. The convolution operation between input neuron and convolution kernels is:

(6) $$x_{k^{\prime }}^{l}=f\left(\sum_{i=1}^{m}\sum_{j=1}^{n}x_{i,j}^{l-1}*w_{k_{1},k_{2},k^{\prime }}^{l}+b^{l}\right)$$

where *m* and *n*, respectively, represent the sizes of the pixels in the height direction and the width direction of the input image; $x_{i,j}^{l-1}$ represents the input of the *l* convolutional layer; $w_{k_{1},k_{2},k^{\prime }}^{l}$ is the weight of the $k^{\prime }$ convolutional kernel at the *l* convolutional layer; the $w_{k_{1},k_{2},k^{\prime }}^{l}$ supports $n\times k_{1}\times k_{2}$ parameters; $k_{1}$ and $k_{2}$ are the kernel size of a filter; n is the number of filters; ∗ is defined convolution operation; $b^{l}$ is the bias of the *l* layer; f is an nonlinear activation function; $x_{k^{\prime }}^{l}$ represents the feature map of the $k^{\prime }$ convolutional kernel in the *l* convolutional layer.

The pooling layer is also called the sub-sampling layer because it involves the division of the input feature map into many small areas of the same length (greater than 1), the calculation of a value for each area, the acquisition of the local optimal value, the arrangement of the calculated values, and the output of a new feature map [35]. The purpose of the pooling layer is to extract features while reducing data dimensions so that it is robust to minor changes in previously learned features. Pooling operations are defined as:

(7) $$x_{k}^{l}=\phi (x_{k}^{l-1})$$

where $x_{k}^{l-1}$ represents the *k* input feature map in the *l* pooling layer; ϕ indicates pooling operation; $x_{k}^{l}$ is the *k* output feature map at *l* pooling layer. In the pooling layer, the numbers of input and output feature maps are the same, but the size of output feature maps is usually reduced to by 50% compared to that of input feature maps. The pooling operations generally includes mean pooling operation and maximum pooling operation. The mean pooling operation is to calculate the average value of each subregion, whereas the max pooling operation is to calculate the max value of each subregion as the output feature.

After the multistage convolutional layer and pooling layer, several fully connected layers are connected. The fully connected layer is used to integrate features from multiple feature maps and map them into a fixed length feature vector. At last, the fully connected layer and the output layer are connected by the classifier, and the learned features are mapped into the sample label space to complete the classification task. Similar to the training process of traditional neural networks, CNN uses back propagation (BP) algorithm to fine tune network parameters.

## 3. Proposed Method Based MPE and MCFCNN

**Figure 3:**
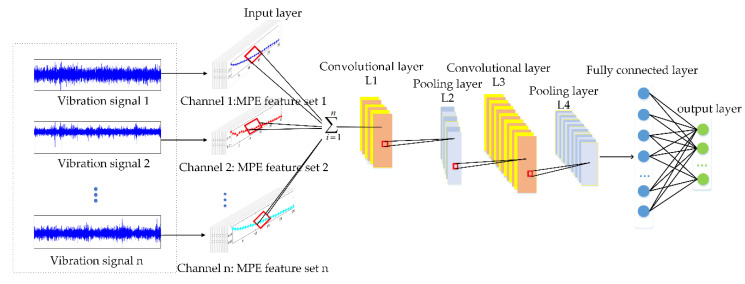
Architecture of multi-channel fusion convolution neural networks (MCFCNN).

The proposed overall MCFCNN framework is shown in Figure 3, which consists of two parts. The first is signal preprocessing. MPE is used to extract the structural information of complex time series from multiple time scales, and the PE value is calculated at each time scale. Then, MCFCNN is constructed to extract features from PE of multiple scales for fault identification. Compared with LeNet model, MCFCNN has multiple channels in the input layer and all channels share a CNN model, thus greatly reducing parameters and training time. Furthermore, unlike image data, the vibration signals of mechanical equipment are one-dimensional data of a time series. Each channel takes one-dimensional series as the input. The convolution layer learns the features of multiple channels at the same time and fuses these learning features. Similar to traditional CNN, MCFCNN model in the training process is to calculate errors according to classification results and real values and feeds back to the whole network through back propagation algorithm to update weights. The fault identification method based on MCFCNN model is described as follows:

Step 1: Condition monitoring at different locations of mechanical equipment through multiple sensors. Assuming that there are n sensors, the input layer of MCFCNN model has n channels. N sensors collect vibration signals for different fault experiments, and get multiple groups of different fault signals, each group of fault signals is represented by $X=\{x_{1},x_{2},\ldots ,x_{m}\}$, m represent the signal length. The health status label corresponding to each signal is represented by Y∈{1,2,…,c} , where c is the total number of fault categories.

Step 2: For each group of vibration signals, sample division is performed, that is, discrete points of vibration signals of length k are intercepted at random positions of each group of signals as a sample, which can be expressed as $X=\left\{x_{i},x_{i+1},\ldots ,x_{i+k-1}\right\}$. Then coarsen the original time series sample X according to the time scale factor s to create coarse-grained time series $y^{\left(s\right)}(j)$. Then calculate the PE value of the coarse-grained sequence $y^{\left(s\right)}(j)$ according to Equations (2)–(5), and finally get MPE value $H_{mpe}$ of time sequence X.

Step 3: The training sample MPE feature of the n sensor is used as the input of the n channel of MCFCNN. The convolutional layer uses the unsupervised learning mode to simultaneously learn the features of each channel and fuses the features extracted from each channel. The multi-channel one-dimensional convolutional calculation process is:

(8) $$x_{k^{\prime }}^{l}=f(\sum_{k=1}^{d}\sum_{j=1}^{m}x_{i,j,k}^{l-1}*w_{k_{1},k_{2},k^{\prime }}^{l}+b^{l})$$

where i=1 and j represent the height and the width of the input data, respectively; d represents the number of input channels; $x_{i,j,k}^{l-1}$ indicates the input of the k channel of the l layer; $w_{k_{1},k_{2},k^{\prime }}^{l}$ is the weight of the $k^{\prime }$ convolution kernel at the l convolutional layer. The convolution kernel is one-dimensional, so $k_{1}=1$, $b^{l}$ is the bias of the *l* layer.

In image classification, the experimental effect of max pooling is better than that of average pooling [36,37]. Max pooling can achieve faster convergence and improve the generalization performance by choosing superior invariants. In the study, the robustness of feature extraction of the convolution layer is improved by non-overlapping maximum pooling, which is expressed as:

(9) $$\begin{array}{l} x_{k}^{l}=\underset{(m-1)p<j<mp}{\max}\left\{x_{i,j,k}^{l-1}\right\}\\ m=1,2,\ldots ,q \end{array}$$

where i=1; p indicates the width of each subregion; q indicates the number of subregions; $x_{i,j,k}^{l-1}$ indicates the input of the k channel of the l layer, $x_{k}^{l}$ indicates the *k* feature mapping of the l−1 layer.

Step 4: MCFCNN is trained with the training set until the loss function converges to a certain degree or the number of iterations is satisfied.

Step 5: The trained MCFCNN is validated with the testing set, and the average testing accuracy and standard deviation are calculated.

## 4. Experimental Validation

**Figure 4:**
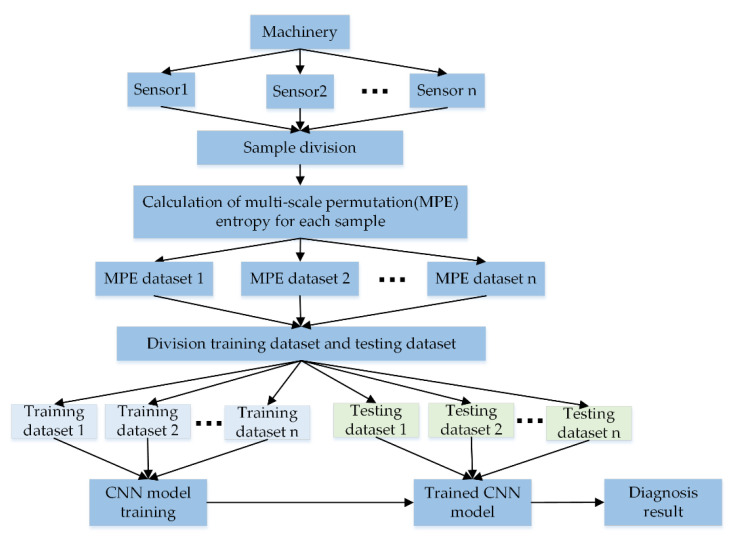
The flowchart of the proposed method.

In order to verify the effectiveness of the proposed method, which was compared with single channel CNN and existing CNN based fusion methods in two fault diagnosis experiments of planetary gearbox and bearing data of Case Western Reserve University (CWRU) [38]. Where single-channel CNN takes single-scale PE value of single sensor as input. Different from other methods, MCFCNN achieves information fusion through multi-channel structure. Firstly, input channels equal to the number of sensors are constructed in the input layer of the network, and these channels correspond to different sensors. Then, the convolutional kernels are used to simultaneously extract features from the signals of different sources and fuse them to provide richer fault information, the flowchart of the proposed method is shown in Figure 4. In order to avoid random sampling errors, 10 groups of tests were carried out in all experiments to ensure the reliability.

### 4.1. Case 1: Fault Diagnosis Experiment of Planetary Gearbox

#### 4.1.1. Experiment and Data Description

**Figure 5:**
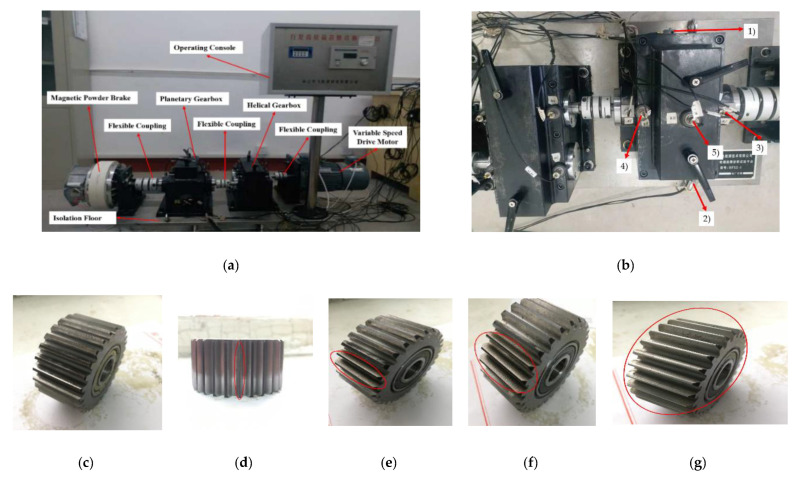
(**a**) Structure of the test rig, (**b**) locations of the five accelerometer sensors, and five states of planetary gear including (**c**) normal planet gear state, (**d**) single tooth worn, (**e**) two teeth worn, (**f**) three teeth worn, and (**g**) all teeth worn.

The planetary gearbox test rig is composed of operating console, a variable speed drive motor, flexible coupling, helical gearbox, planetary gearbox, a magnetic powder brake, and isolation floor. The motor is connected with the helical gearbox through the flexible coupling [39]. The planetary gearbox and the helical gearbox are connected with the flexible coupling and finally the planet gearbox is connected with the magnetic powder brake. The operating console is used to adjust the frequency of the motor (0–60 Hz) and the load of the magnetic powder brake (0–100 N·m). The output power of the motor is transmitted to the planetary gearbox through bearings and helical gearboxes in turn. The structure of the test rig is shown in Figure 5a. The sun gear in the planetary gearbox is surrounded by a fixed gear ring and three rotating planet gears and transmits torque to planetary gears and the planetary carrier [40]. The carrier then transmits torque to the output shaft. The details of the planetary gearbox are given in Table 1. Two 3-axis accelerometers and three 1-axis accelerometers are mounted upon planetary gearbox case to acquire vibration signals, the position of the accelerometer is shown in Figure 5b. The symbols of “(1), (2), (3), (4), and (5)” indicate the monitoring positions of acceleration sensors on the gearbox. Among them, (1) and (2) were 3-axis sensors.

Most of the previous studies focused on fault diagnosis of fixed-axis transmission or relative stationary components such as sun gear, inner gear rings and planetary carriers [41], but condition monitoring and fault diagnosis of planet gears were seldom reported. In the experiment, five states of planetary gear faults with different wear degrees were designed: normal state, single tooth worn (stw), two teeth worn (ttw), three teeth worn (thtw), and all teeth worn (atw). The damaged planetary gears are shown in Figure 5c–g. For each state, the loads of motor were 0.3 horsepower (hp), 0.5 hp, and 1 hp, respectively. All vibration signals were collected at a sampling frequency of 20.48 kHz and sampling time was 30 s. According to the structural parameters and speed of the planetary gearbox, the characteristic frequency of the distributed faults of each gear can be calculated, as shown in Table 2.

**Figure 6:**
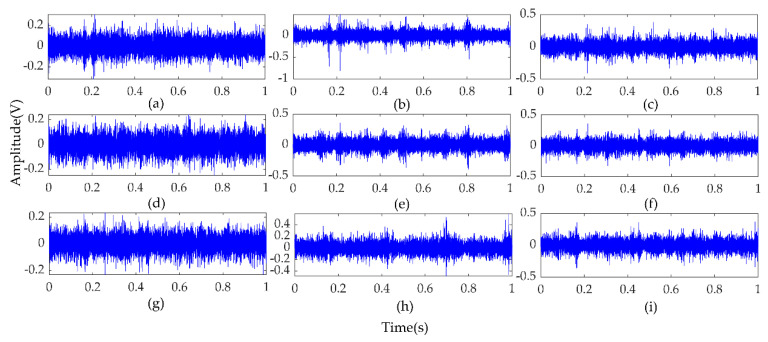
Raw vibration signals of the planet gear with atw monitored in nine directions under a load of 0.5 hp. (**a**–**c**) Signals detected with Sensor 1 in the directions of x, y and z axes; (**d**–**f**) Signals detected with Sensor 2 in the directions of x, y, and z axes; (**g**–**i**) Signals detected with Sensor 3, Sensor 4, and Sensor 5 in the x-axis direction.

**Figure 7:**
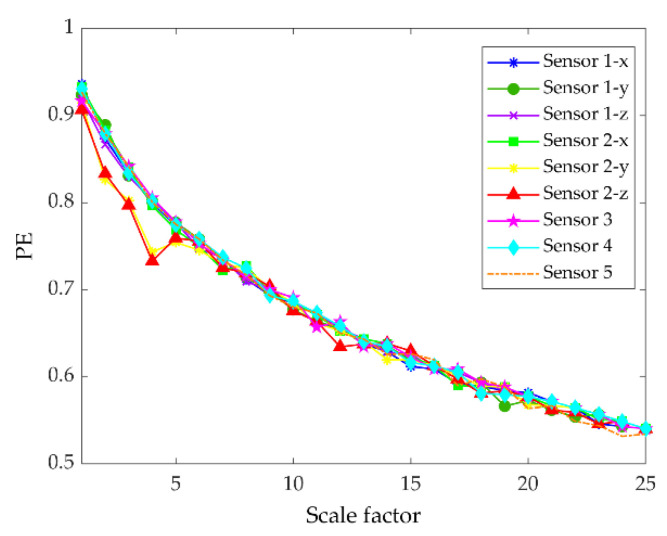
Multiscale permutation entropy of one sample with atw monitored in nine directions under load of 0.5 hp.

In the experiment, each sample contains 1024 data points, and the faulty planetary gear has no mesh within the sample length. Therefore, two 3-axis accelerometers and three 1-axis accelerometers were used to monitor vibration signals in nine directions, and the total number of samples for each state was 5400. Raw vibration signals of the planet gear with atw monitored in nine directions under a load of 0.5 hp are shown in Figure 6. We can see that the signals monitored at different positions contained different information. Combined with multi-sensor data, these signals could provide more information for fault diagnosis. Then, setting the coarse-grained scale factor s=25, embedding dimension m=6, and time delay λ=1 to obtain 5400∗25 MPE feature sets of the sample. The 25 scale MPE corresponding to the vibration signal of Figure 6 is shown in Figure 7. For each load, 480 samples in each direction were randomly selected as the training set. The remaining 120 samples were the testing set. Table 3 provides the three datasets under different loads. 

#### 4.1.2. Model Design

The number of channels in the input layer of MCFCNN model can be set according to the number of sensors used in the experiment. In the planetary gearbox experiment, five groups of sensors were used to monitor vibration signals in nine directions, so the number of channels in the input layer is 9. There were 10 convolutional kernels and 20 convolutional kernels in the two convolutional layers. The convolutional kernels of two convolution layers were set to be 1 × 3. The moving step was set to be 1 and the ratio of max pooling was set to be 1 × 2. At the fully connected layer, the number of neurons was set to be 200 and Softmax regression was adopted as a classifier. Details of the parameters of the MCFCNN model are given in Table 4. The structure and parameter initialization of single-channel CNN used for comparison are the same as those of MCFCNN.

#### 4.1.3. Comparison between Multi-Channel Fusion Convolution Neural Networks (MCFCNN) and Single-Channel Convolutional Neural Networks (CNN)

**Figure 8:**
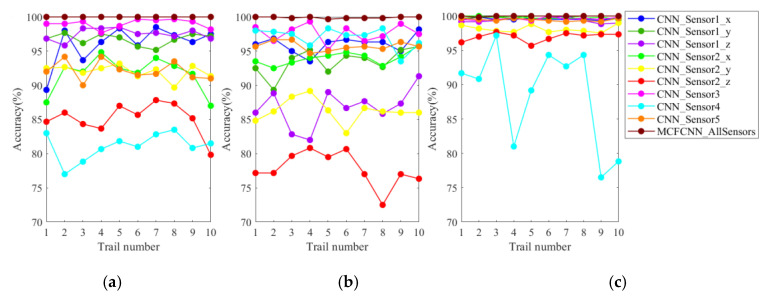
Diagnostic accuracy of ten experiments with single-channel convolutional neural networks (CNN) based on single-sensor data and MCFCNN based on multi-sensor data on test sets A (**a**), B (**b**), and C (**c**), respectively.

To verify MCFCNN in gearbox fault diagnosis, the proposed method was compared with single-channel CNN. The two methods were tested through ten trials with three datasets under three loads, respectively. The diagnostic accuracy of each experiment is shown in Figure 8. The diagnostic accuracy of the MCFCNN was higher and not affected by load. Table 5 lists the average testing accuracy and standard deviation of ten trials and average training time of the two methods. The average diagnostic accuracy of MCFCNN under different loads was between 99.90% and 100% and that of single-channel CNN was between 81.10% and 99.58%. The average testing accuracy of single-channel CNN based on X-axis orientation of Sensor 1 and Sensor 5 was between 96.12% and 99.58%. Although they had achieved the acceptable testing accuracy, the standard deviation of single-channel CNN was much larger than that of MCFCNN model. In addition, compared with single-channel CNN, the multi-channel structure of MCFCNN can improve the accuracy of diagnosis with little impact on training time.

**Figure 9:**
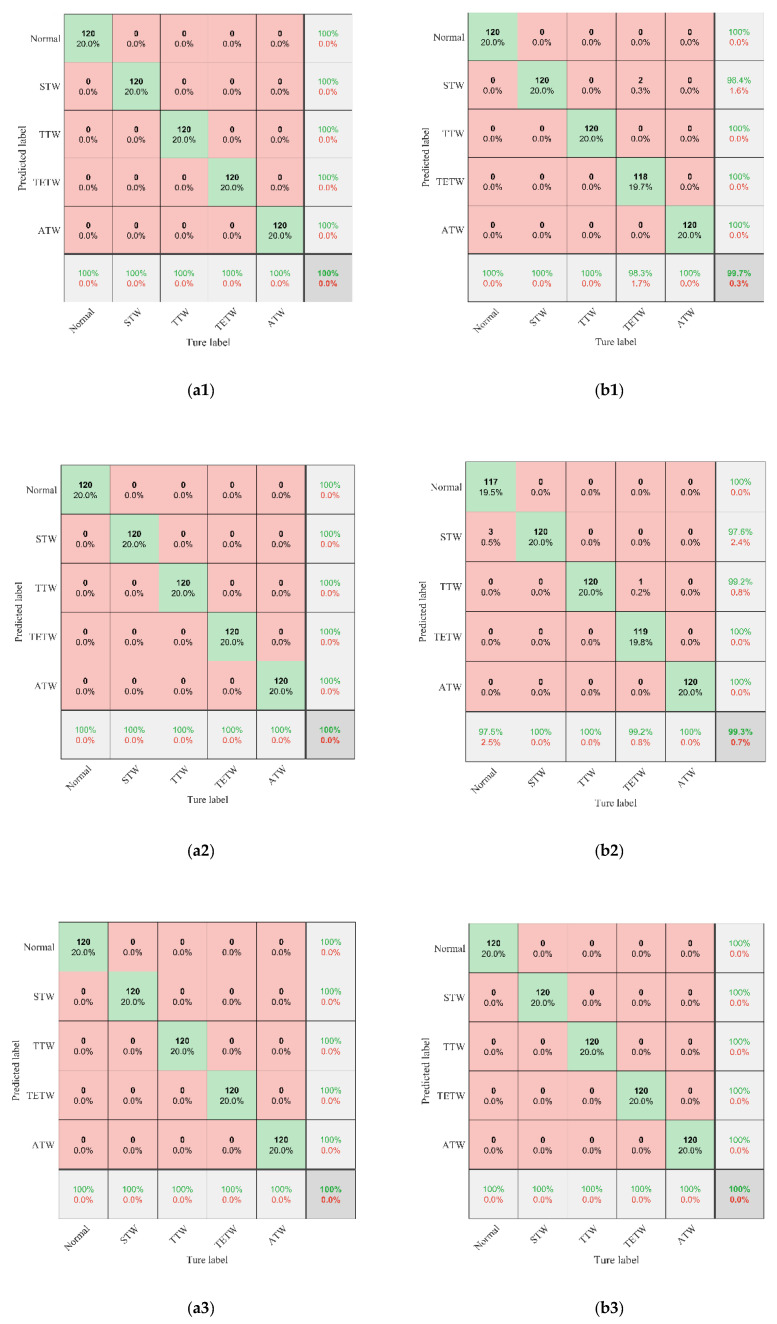
Confusion matrices of five wear fault conditions. (**a1**–**a3**) are the confusion matrices of MCFCNN method in testing datasets A, B, and C, respectively; (**b1**–**b3**) are the confusion matrices of single-channel CNN method in testing datasets A, B, and C, respectively.

To further assess the classification performance of MCFCNN, the diagnostic reliability of two diagnostic methods for each fault state of planetary gear was obtained with the confusion matrix. The confusion matrices of five fault states of MCFCNN and single-channel CNN are shown in Figure 9. The column and row of the confusion matrix represents, respectively, the prediction category and the real category. Green data at the last raw and the last column, respectively, indicate the precision of each fault state and the recall of each fault. The diagnosis results of each fault state can be obtained from the confusion matrix. Figure 9a1–a3 show the confusion matrices of MCFCNN for fault identification of datasets A, B, and C, and Figure 9b1–b3 show the confusion results of single-channel CNN for the fault identification of the datasets A, B, and C. As can be seen from Figure 9, the best diagnostic results of single-channel CNN still contain misdiagnosis. In the testing dataset A, the trained single-channel CNN misdiagnosed two samples with thtw as stw and had an accuracy rate of 99.7% and a total error of 0.3%. In the testing dataset B, one sample with thtw was misdiagnosed as the sample with ttw and three normal samples were misdiagnosed as stw and the trained CNN had an accuracy rate of 99.3% and a total error of 0.7%. MCFCNN can well diagnose different levels of wear faults, and the diagnostic accuracy reaches 100%.

**Figure 10:**
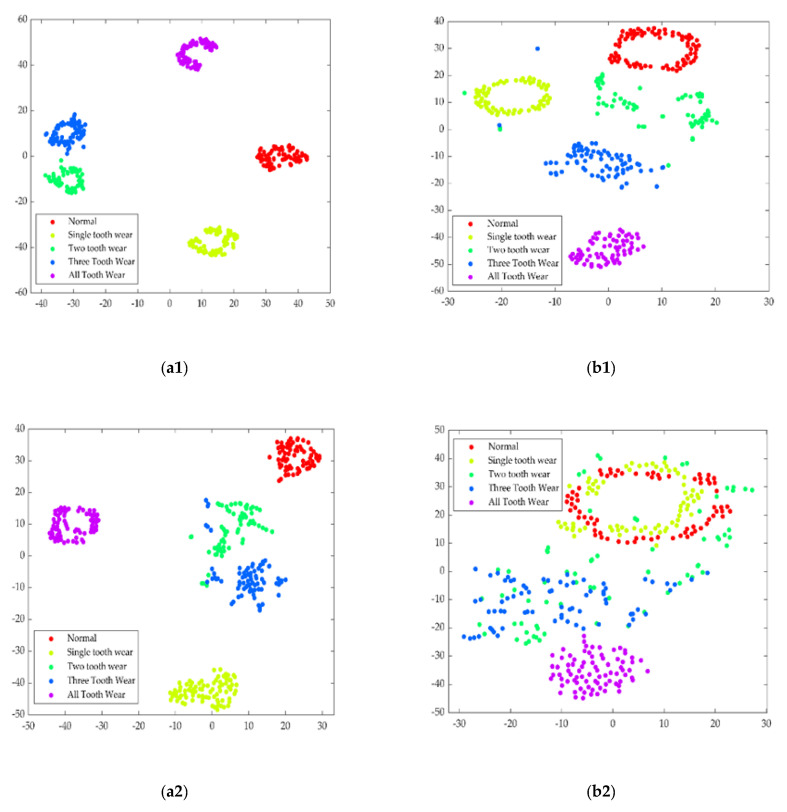

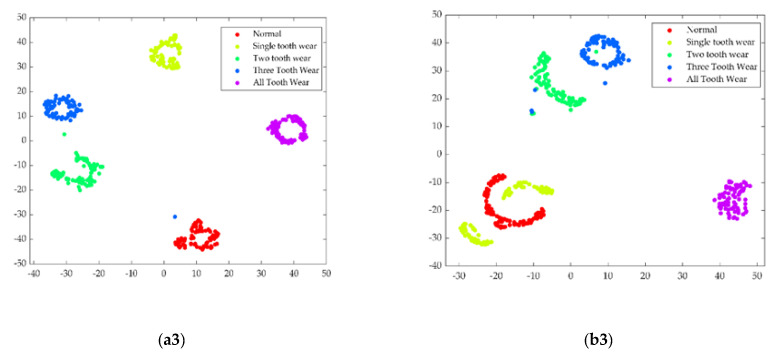
T-SNE visualization of features learned in the fully connected layer: (**a1**–**c1**) indicate the features of MCFCNN from testing datasets A, B, and C; (**a2**–**c2**) indicate the features of single-channel CNN from testing datasets A, B, and C.

To verify the feature learning ability of the method, the features in the fully connected layers of MCFCNN and single-channel CNN were visualized by t-distributed stochastic neighbor embedding (t-SNE). When MCFCNN and single-channel CNN were used to diagnose the testing datasets A, B, and C, the features obtained by the fully connected layer were reduced to 2D by the T-SNE technique to observe the classification effect. As shown in Figure 10, the fully connected layers of the two models were visualized by the t-SNE [42]. Each point indicates a sample and the axis indicates the t-SNE dimension.

In terms of the feature distribution of the fully connected layer, MCFCNN had the good feature separation capability and classification performance. Multi-channel data fusion and feature fusion could be well clustered into categories, and not adversely affected by bad signals. It can be judged that MCFCNN with multi-sensor data as the input can effectively delete redundant information in the process of feature learning and improve the classification accuracy. Single-channel CNN showed the good feature separation capability under testing datasets A and C, but the features of two tooth worn state and three tooth worn state were mixed together. In the testing dataset B, except that the feature of all tooth worn state was clustered together, the features of three tooth worn state and two tooth worn state overlapped and the features of normal state and single teeth worn state overlapped, it was difficult to differentiate the fault of two tooth worn from the fault of three tooth wear or the normal state from the fault of single teeth worn. In addition, fault size or other properties such as load may lead to the feature overlapping.

#### 4.1.4. Comparison between MCFCNN and Other Fusion Methods Based on CNN

**Figure 11:**
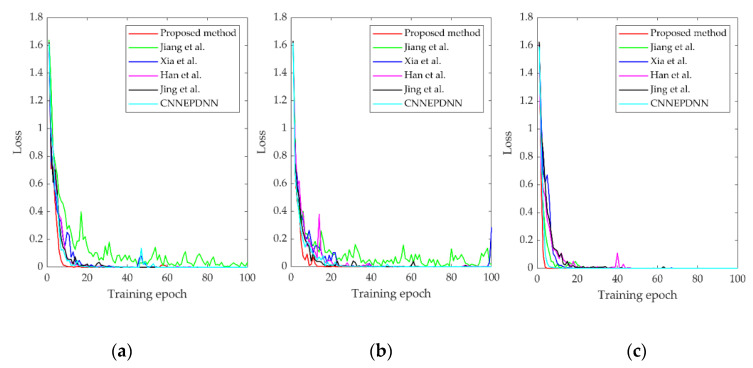
(**a**–**c**). An example of the training loss of comparative methods under the training sets A, B, and C.

In order to further prove the stability and superiority of MCFCNN, it is compared with other fusion methods [13,14,16,17] based CNN and our former model CNNEPDNN [18]. On the basis of CNN, Jiang et al. [13] and Xia et al. [14] used data splicing to realize data fusion. Jiang et al. [13] spliced the vibration signal, acoustic signal, current signal and instantaneous angular speed signal into a one-dimensional matrix, Xia et al. [14] spliced the vibration signal collected by multiple sensors into a two-dimensional matrix. Then used the obtained matrix as the input of the CNN. Han et al. [16] proposed DECNN, which integrates multiple parallel CNN through a dynamic ensemble layer which assigns weight to each branch to achieve the fusion of multi branch features. Multi-level wavelet coefficients matrixes (MWCMs) were used as the input of each branch. Jiang et al. [17] proposed a similar MSCNN to address the problem of multi-scale feature extraction. First, the vibration signal was coarse-grained with different scale factors, and then different coarse-grained sequences were fed into the branches of MSCNN. In [18], we constructed a CNNPEDNN model for DNN parallel ensemble CNN based on feature fusion. The vibration signals and time domain statistical features were used as the input of the two branches of the model. The diagnostic results of various methods are shown in Figure 11 and other performance analyses are listed in Table 6.

Under three datasets, MCFCNN had the better classification performance than other methods (Table 6 and Figure 11). Our former CNNEPDNN model is based on the feature fusion of single sensor data. Without the help of multi-sensor data and multi-scale analysis, the performance of the CNNEPDNN model decreased. Jing’s method and Xia’s method took all sensor data as the input, but MCFCNN adopted the multi-channel structure and could effectively remove redundant information and improve diagnosis results and stability. Compared with Han’s method and Jiang’s method, multi-channel structure of MCFCNN method is simple and easy. Multi-channel shares a CNN model, which greatly reduces the training parameters, and accelerates the convergence of loss function. In the experiment, the average training time of MCFCNN method is 0.1 s, which meets actual application requirements.

#### 4.1.5. Load Adaptability Verification

In practical engineering application, the load of rotating machinery always changes, and the fault diagnosis model needs to adapt to different load conditions. In order to verify the load adaptability of the proposed method, the training datasets A, B, and C are used to train the model, and the other two testing datasets different from the training set are used to test the trained model. For example, the model is trained on training dataset A, and tested on testing dataset B, which is represented by symbol “A→ B”. Therefore, there are six combinations between the training set and the test set, each training dataset contains 480 samples and the test dataset contains 120 samples. It can be observed from Table 5 that under three kinds of loads, the average testing accuracy of single channel CNN based on X-axis orientation of Sensor 1 and Sensor3 is better than that of other position sensors. Therefore, in this experiment, PE values of vibration signals of these two sensors are used as sample dataset to test the load adaptability of single channel CNN model, and the other CNN based fusion methods [13,14,16,17,18] is also investigated. The experiment result are shown in Table 7.

Compared with Table 5 and Table 6, the average test accuracy of each method decreased significantly. We suppose that due to the complexity of the signal transmission path of the planetary gearbox, the features extracted by the model are not sensitive to load changes. In the case of “A→B” and “B→A”, all methods achieve high accuracy, which means that the features of datasets A and B are more similar than others. Overall, compared with other methods, MCFCNN achieves the highest average accuracy, which proves that MCFCNN has strong load adaptability.

### 4.2. Case 2: Fault Diagnosis Experiment of Rolling Bearing

It is difficult to verify the performance of the proposed method with a single dataset, so the proposed method is also tested on CWRU’s rolling bearing fault dataset. CWRU dataset is the world recognized standard dataset for bearing fault diagnosis. In the field of fault diagnosis based on deep learning, the test data of the two most cited papers [43,44] are all from the CWRU bearing dataset. In order to evaluate the superiority of the proposed method, the most objective way is to use a third-party standard dataset to compare with current mainstream algorithms. Therefore, this paper uses CWRU bearing dataset.

#### 4.2.1. Experiment and Data Description

**Figure 12:**
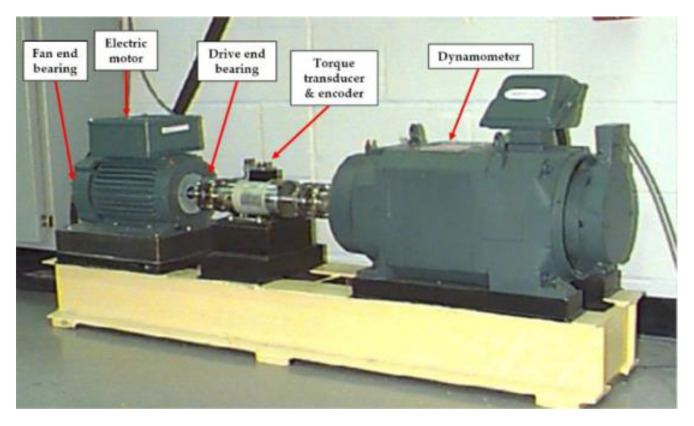
Experimental platform for acquiring vibration signals from rolling bearings.

As shown in Figure 12, the rolling bearing fault experiment platform of CWRU is composed of 2 horsepower (1.5 kW) motor, torque sensor, acceleration sensor, power tester, etc. The rolling bearing models at the drive end and fan end are 6205-2rs JEM SKF and 6203, respectively. The single point faults with diameters of 7, 14, and 21 mils were manufactured on the inner raceway, outer raceway, and rolling body of normal bearing by electro-discharge machining (EDM). In the case of inner raceway fault, two acceleration sensors were installed on the 12 o’clock position above the motor drive end (DE) and fan end (FE) through a magnetic base. In the case of outer raceway fault and rolling element fault, in addition to the drive end and fan end, sensor was installed on the motor supporting plate (SBP) to collect signals. The vibration signals were collected by 16 channel DAT recorder, and the sampling frequency was 12 kHz.

**Figure 13:**
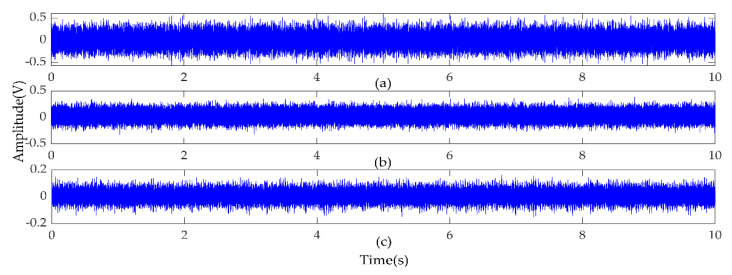
Vibration signal of rolling element fault with fault size of 7 mils. (**a**–**c**) are vibration signals monitored on the drive end, fan end and supporting base plate, respectively.

**Figure 14:**
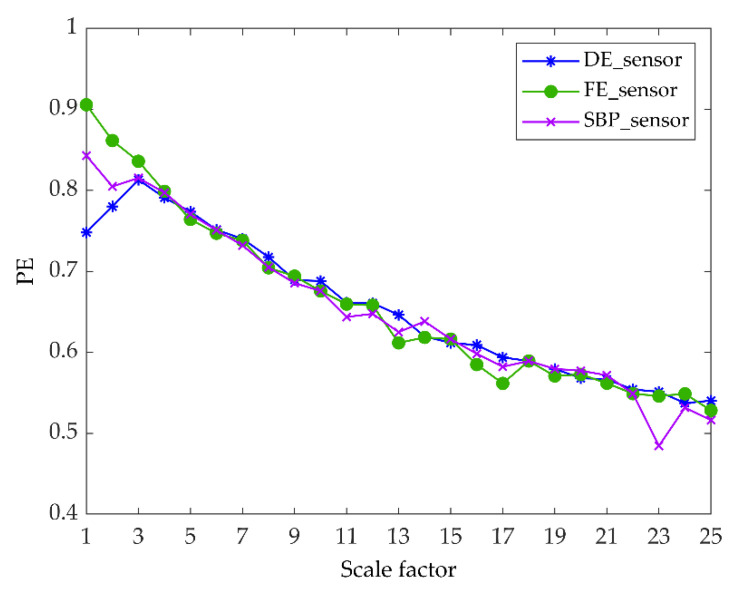
Multi-scale permutation entropy (MPE) value of three direction samples.

Due to the different number of sensors, this paper studies 6 classes of faults with different severity on the rolling element and outer raceway of the drive end rolling bearing when the motor load is 0, 2, 3 horsepower (hp). Figure 13 shows the time-domain waveform of the 10 s vibration signal collected by the sensors at the drive end, fan end and motor support base plate when the motor load is 2 hp and the fault diameter of the rolling element is 7 mils. The vibration signals of each type of fault state monitored by the sensor are divided into samples of the same length, each sample length is 1024, and the number of samples of each health state is 118. Therefore, the samples at each load generate datasets A, B, and C, and each dataset is divided into training set and test set according to 80% and 20% percentages. A detailed description of the experimental data is given in Table 8. According to the MPE parameter setting in Experiment 1, the MPE value of each sample is calculated, and the MPE feature set of size 118 × 25 is obtained. The result of MPE corresponding to the vibration signal of Figure 13 is shown in Figure 14.

#### 4.2.2. Model Designing

Three groups of sensors were used to monitor the vibration signals in three directions in the rolling bearing experiment of CWRU. Therefore, the number of channels in the input layer of MCFCNN model is 3, and other parameters are the same as those in Experiment 1.

#### 4.2.3. Comparison between MCFCNN and Single-Channel CNN

**Figure 15:**
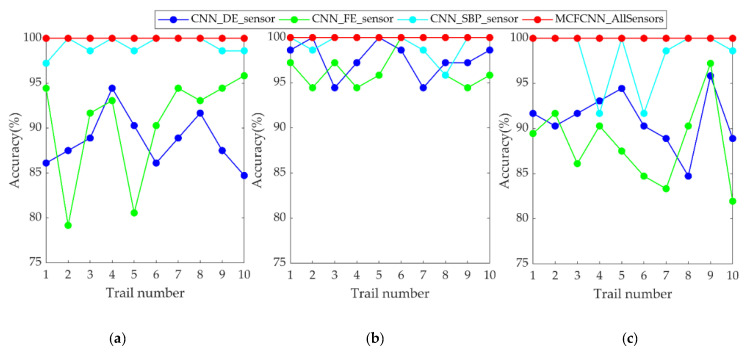
Diagnostic accuracy of ten experiments with single-channel CNN based on single-sensor data and MCFCNN based on multi-sensor data on test sets A (**a**), B (**b**), and C (**c**), respectively.

MCFCNN model and single channel CNN model are used for rolling bearing fault diagnosis. MCFCNN model simultaneously takes the MPE value of DE, FE, and SBP sensor data as input, and single channel CNN takes PE value of single sensor as input. The diagnostic accuracy of the two models in ten experiments on three datasets under three loads are shown in Figure 15. It can be seen from the figure that the test accuracy of MCFCNN model is always better than that of single channel CNN model for each load data. Table 9 gives the average test accuracy, standard deviation, and average training time of the two models. The average test accuracy of single-channel CNN on SBP sensors reached 99.17%, 99.31%, and 98.06%, while the highest average test accuracy on DE and FE sensors was 97.64% and 96.39%, respectively. In contrast, the average test accuracy of MCFCNN model is 100%, which further illustrates that the proposed method can improve the accuracy of diagnosis by fusing MPE features of multi-sensor vibration signals.

#### 4.2.4. Comparison between MCFCNN and Other Fusion Methods Based on CNN

The model used for comparison is the same as the planetary gearbox fault diagnosis experiment, and the experimental results are shown in Table 10. The results show that the average diagnostic accuracy of MCFCNN model is 100%, the average training time of MCFCNN model is 0.02 s, and the performance of MCFCNN model is better than other fusion models.

#### 4.2.5. Load Adaptability Verification

The load adaptability of the proposed method is verified on the rolling bearing data. During the experiment, the models trained on different training datasets A, B, and C are tested on different testing datasets. Each training dataset and testing dataset contains 94 and 24 samples, Table 11 shows the test accuracy results of the comparison methods. Note: as can be seen from Table 9, the diagnosis results of single channel CNN on SBP sensor data are better, so in this experiment, the sample dataset of single channel CNN comes from the PE features of the vibration signals of SBP sensor.

It can be seen from the results that under different loads, the proposed method achieves higher accuracy than other methods, but compared with Table 9 and Table 10, the average test accuracy of each method is significantly reduced. However, in the case of “B→A”, the average test accuracy of the proposed method is 76.11%, which is higher than other method, but the load adaptive performance is not good. In addition, we also found that there is a 20.19% difference in the test accuracy of the MCFCNN model between “A→B” and “B→ A” cases. This is because the MPE features of the fusion DE sensor and the FE sensor is helpful for the “A→B” case, but not for the “B→A” case. In the case of “B→C” and “C→B”, all methods have achieved higher accuracy, which means that the characteristics of datasets B and C are relatively similar.

## 5. Conclusions

In this paper, a fault diagnosis method based on MCFCNN and MPE is proposed for the fault diagnosis of rotating machinery. Firstly, the permutation entropy across 25 scales is extracted from the vibration signal to represent the fault information of the signals on multiple timescales. The extracted MPE features are then input to MCFCNN for information fusion and fault identification. The novelty of MCFCNN is that several channels are built for the input layer, which correspond to different sensors. Sharing a CNN structure between channels. MCFCNN realizes end-to-end multi-source information fusion and classification without human intervention. The proposed method was verified by the experimental data of the planetary gearbox and rolling bearing. Experimental results show that the proposed method can effectively identify faults with different severity of gearbox and rolling bearing. Through comparative research with single-channel CNN and other CNN-based fusion methods, the proposed method has obvious superiority in learning ability, loss function convergence speed, training time, fault recognition, and load adaptability.

In the next work, we will test the proposed method under more conditions. Furthermore, in terms of load adaptation, there is still the possibility of improving the fault identification rate, so we will consider using different types of sensors to fuse and optimize the network structure, so as to achieve better fault diagnosis performance.

## Figures and Tables

**Table 1:** Parameters of the planetary gearbox.

Gears	Tooth Number
Sun gear	18
Planetary gear (number)	27(3)
Ring carrier	72

**Table 2:** Planetary gearbox characteristic frequency.

Load/Speed	Meshing Frequency	Rotating Frequency			Fault Characteristics Frequencies		
		Sun Gear	Planetary Gear	Ring Carrier	Sun Gear	Planetary Gear	Ring Carrier
0.3 hp/40 HZ	257.1 HZ	14.29 HZ	9.524 HZ	2.857 HZ	14.286 HZ	9.523 HZ	3.571 HZ
0.5 hp/50 HZ	321.4 HZ	17.86 HZ	11.9 HZ	17.857 HZ	17.857 HZ	11.905 HZ	4.464 HZ
1 hp/20 HZ	128.6 HZ	7.143 HZ	4.762 HZ	1.429 HZ	7.143 HZ	4.762 HZ	1.786 HZ

**Table 3:** Experimental datasets.

Planetary Gear States		Normal			stw			ttw	thtw	atw
Class Label		0			1			2	3	4
Dataset A	Training	480	480	480	480	480	480	480	480	480
	Testing	120	120	120	120	120	120	120	120	120
Dataset B	Training	480	480	480	480	480	480	480	480	480
	Testing	120	120	120	120	120	120	120	120	120
Dataset C	Training	480	480	480	480	480	480	480	480	480
	Testing	120	120	120	120	120	120	120	480	480

**Table 4:** Parameters of the MCFCNN model.

MCFCNN	Structure/Training Settings	Parameters
Structure of MCFCNN	Number of channels of input layer	9
	Number and size of convolutional kernels in two convolutional layers, and the moving step of the convolutional kernel	20, 40, 1 × 3, 1
	Stride of the 2 max-pooling layers	1 × 2
	Neuron numbers of the fully connected layer	100
Input of each channel in input layer	Sample size of Sensor 1 in the x-axis direction	1 × 25
	Sample size of Sensor 1 in the y-axis direction	1 × 25
	Sample size of Sensor 1 in the z-axis direction	1 × 25
	Sample size of Sensor 2 in the x-axis direction	1 × 25
	Sample size of Sensor 2 in the y-axis direction	1 × 25
	Sample size of Sensor 2 in the z-axis direction	1 × 25
	Sample size of Sensor 3	1 × 25
	Sample size of Sensor 4	1 × 25
	Sample size of Sensor 5	1 × 25
Training settings	Mini-batch size	100
	Learning ratio	0.0015
	Total epochs	100

**Table 5:** Average testing accuracy, standard deviation, and model training average time of single-channel CNN based on single-sensor data and MCFCNN based on multi-sensor data.

Inputs	Methods	Average Testing Accuracy ± Standard Deviation (%)			Average Training Time (s)
		A	B	C	
Sensor 1-x	CNN	96.11 ± 2.78	96.02 ± 1.26	99.57 ± 0.16	0.08
Sensor 1-y	CNN	96.74 ± 0.83	93.48 ± 1.91	99.88 ± 0.14	0.11
Sensor 1-z	CNN	97.50 ± 0.85	86.75 ± 2.81	99.37 ± 0.35	0.08
Sensor 2-x	CNN	91.68 ± 2.53	93.97 ± 0.98	99.78 ± 0.18	0.06
Sensor 2-y	CNN	92.02 ± 1.03	86.27 ± 1.69	98.12 ± 0.53	0.04
Sensor 2-z	CNN	85.15 ± 2.30	77.78 ± 2.50	96.97 ± 0.63	0.07
Sensor 3	CNN	99.00 ± 0.66	97.62 ± 1.30	99.58 ± 0.21	0.06
Sensor 4	CNN	81.10 ± 1.98	97.02 ± 1.50	88.65 ± 7.23	0.07
Sensor 5	CNN	92.15 ± 1.40	95.72 ± 0.67	99.43 ± 0.20	0.08
Sensor 1-x–Sensor 5	MCFCNN	100	99.90 ± 0.12	99.98 ± 0.05	0.10

**Table 6:** Performance analysis of various methods.

Methods	Average Testing Accuracy ± Standard Deviation (%)			Average Training Time (s)	Number of Parameters
	A	B	C		
Jing et al. [13]	92.65 ± 3.49	93.83 ± 1.84	98.71 ± 0.67	24.6(CPU)	133,517
Xia et al. [14]	84.35 ± 3.81	77.92 ± 6.35	87.11 ± 3.91	27(CPU)	142,274
Han et al. [16]	92.11 ± 2.02	87.59 ± 3.21	94.65 ± 2.09	17(GPU)	218,138
Jiang et al. [17]	91.91 ± 2.80	87.34 ± 2.69	95.41 ± 1.67	15(GPU)	135,454
CNNPEDNN [18]	89.08 ± 1.15	85.44 ± 1.28	90.08 ± 1.34	0.13	132,981
MCFCNN	100	99.90 ± 0.12	99.98 ± 0.05	0.1(CPU)	115,942

**Table 7:** Different combinations of training dataset and testing dataset of planetary gearbox.

Input	Method	A→B	A→C	B→A	B→C	C→A	C→B	Average
PE features of sensor 1	CNN	51.81	41.37	58.47	42.83	36.56	31.82	43.81
PE features of sensor 3	CNN	62.82	48.90	68.07	47.93	34.92	32.24	49.15
Vibration signals	Jing et al. [13]	67.70	47.95	63.84	46.65	48.82	52.91	54.65
Vibration signals	Xia et al. [14]	57.53	43.11	56.80	33.33	46.14	31.74	44.78
MWCMs	Han et al. [16]	62.10	54.09	67.16	43.10	45.54	46.82	51.47
Vibration signals	Jiang et al. [17]	73.65	62.35	70.58	50.26	59.87	48.34	60.84
Vibration signals and time domain statistical features	CNNEPDNN [18]	60.07	51.50	63.45	35.67	47.67	45.34	50.61
MPE features	MCFCNN	88.20	75.28	92.75	77.33	78.20	74.83	81.10

**Table 8:** Experimental datasets.

Fault Location		Rolling Element			Outer Raceway		
Fault size (mils)		7	14	21	7	14	21
Class label		0	1	2	3	4	5
Dataset A	Training	94	94	94	94	94	94
	Testing	24	24	24	24	24	24
Dataset B	Training	94	94	94	94	94	94
	Testing	24	24	24	24	24	24
Dataset C	Training	94	94	94	94	94	94
	Testing	24	24	24	24	24	24

**Table 9:** Average testing accuracy, standard deviation, and model training average time of single-channel CNN based on single-sensor data and MCFCNN based on multi-sensor data.

Inputs	Methods	Average Testing Accuracy ± Standard Deviation (%)			Average Training Time (s)
		A	B	C	
DE_Sensor	CNN	88.61 ± 2.91	97.64 ± 1.97	90.97 ± 3.16	0.04
FE_Sensor	CNN	90.70 ± 5.93	96.39 ± 1.88	88.25 ± 4.51	0.04
SBP_Sensor	CNN	99.17 ± 0. 0.97	99.31 ± 1.35	98.06 ± 3.41	0.04
All Sensors	MCFCNN	100	100	100	0.02

**Table 10:** Performance analysis of various methods.

Methods	Average Testing Accuracy ± Standard Deviation (%)			Average Training Time (s)	Number of Parameters
	A	B	C		
Jing et al. [13]	99.28 ± 0.23	99.65 ± 0.30	98.65 ± 0.43	5(CPU)	133,517
Xia et al. [14]	99.87 ± 0.35	99.41 ± 0.33	99.75 ± 0.21	7(CPU)	142,274
Han et al. [16]	98.78 ± 0.71	99.11 ± 0.63	98.61 ± 0.57	10(GPU)	218,138
Jiang et al. [17]	99.05 ± 0.39	98.84 ± 0.68	99.59 ± 0.46	10(GPU)	135,454
CNNEPDNN [18]	95.76 ± 0.70	97.62 ± 0.42	98.10 ± 0.46	0.14(CPU)	132,981
MCFCNN	100	100	100	0.02 (CPU)	115,942

**Table 11:** Different combinations of training dataset and testing dataset of rolling bearing.

Input Data	Method	A→B	A→C	B→A	B→C	C→A	C→B	Average
PE features	CNN	56.02	60.42	53.17	84.52	59.32	90.90	67.39
Vibration signals	Jing et al. [13]	65.51	64.63	60.00	82.18	67.22	88.33	71.31
Vibration signals	Xia et al. [14]	70.47	62.33	64.07	81.31	68.60	85.69	72.08
MWCMs	Han et al. [16]	74.53	65.60	62.86	80.45	68.27	83.45	72.53
Vibration signals	Jiang et al. [17]	83.73	89.51	75.34	93.25	85.42	95.14	87.07
Vibration signals and time domain statistical features	CNNEPDNN [18]	58.29	76.54	59.82	83.10	63.71	88.59	71.68
MPE features	MCFCNN	96.30	93.61	76.11	97.06	94.05	98.75	92.65
